# Association between gamma-glutamyl transferase levels and the retinal age gap

**DOI:** 10.3389/fphys.2025.1601093

**Published:** 2025-08-26

**Authors:** Kai Yang, Xiaoxuan Zhu, Ziyu Li, Wei Lian, Jinxia Yan, Shasha Ding, Zhenqing Wang, Yudie Wang, Jiaqi Ai, Zhengyang Guo, Binbin Su, Jia Qu, Fan Lu, Lele Cui, Ming Li

**Affiliations:** National Clinical Research Center for Ocular Diseases, Eye Hospital, Wenzhou Medical University, Wenzhou, Zhejiang, China

**Keywords:** retinal age gap, gamma-glutamyl transferase, foundation model, aging acceleration, non-invasive screening

## Abstract

**Background:**

To develop a retinal age prediction model based on a foundation model using fundus images and to determine the association between gamma-glutamyl transferase (GGT) levels and the retinal age gap.

**Methods:**

A total of 36,044 fundus images with reasonable quality from 9,752 participants in the Jidong Eye Cohort Study were included in this study. Of these images, 8,869 fundus images from 3,010 healthy individuals were used to train and validate the model based on the foundation model RETFound for age prediction using 10-fold cross-validation. A total of 4,081 fundus images from 4,081 participants who were enrolled from May to October 2023 had available GGT data, and these images were used to investigate the association between GGT levels and the retinal age gap.

**Results:**

The trained model in this study achieved excellent performance, with a mean absolute error (MAE) of 2.42 ± 0.08 years. The mean age of the participants in the analysis dataset was 43.7 ± 10.4 years, and 1987 (48.7%) participants were women. The multivariable βs and 95% confidence intervals (CIs) of the retinal age gap in the second, third, and fourth GGT quartiles compared with the lowest GGT quartiles were 0.42 (0.08–0.77), 0.54 (0.15–0.92), and 0.72 (0.29–1.14) (P for trend = 0.001), respectively, in the fully adjusted model (adjusted for age, sex, current smoking status, current drinking status, body mass index, hypertension, diabetes, dyslipidemia, and serum uric acid).

**Conclusion:**

Increased GGT levels were significantly associated with accelerated retinal aging as quantified by the retinal age gap. Our findings indicate that elevated GGT levels may have an adverse effect on the aging process.

## Introduction

Gamma-glutamyl transferase (GGT), which is a ubiquitous enzyme that is critical for glutathione metabolism and oxidative stress regulation, has increasingly been identified as a biomarker in addition to its traditional role in hepatobiliary health (Emdin et al., 2005; Kunutsor, 2016; Brennan et al., 2022). Elevated GGT levels are closely correlated with systemic oxidative damage, chronic inflammation, and metabolic dysfunction, all of which play critical roles in the progression of aging (Ali et al., 2016; Corti et al., 2020; Chiyanika et al., 2025). Several studies have demonstrated that higher GGT levels are associated with various age-related chronic diseases, including cardiovascular disease, cognitive impairment, and all-cause/disease-specific mortality, suggesting its profound relevance for the aging process (Breitling et al., 2011; Jeon et al., 2020; Tang et al., 2021; Cho et al., 2023). Furthermore, emerging evidence suggests that GGT may mediate the effects of modifiable lifestyle factors, such as diet, sleep, and cardiovascular risk profiles, on aging (Liu et al., 2024, pp. 2005–2018; Wang X. et al., 2024; Xu et al., 2024). However, direct investigations into the association between GGT and biological aging remain scarce, highlighting a critical gap in understanding the use of GGT as a systemic aging marker.

The retina is an ideal window for assessing systemic aging (Zhu et al., 2023; Tan et al., 2024; Wang et al., 2025), given its shared embryological origins and microvascular features with vital organs (e.g., the brain, heart, and kidneys) (Patton et al., 2005; Flammer et al., 2013; Wong et al., 2014; Li et al., 2023); thus, retinal alterations reliably reflect systemic circulatory health and neurodegenerative processes. Importantly, retinal imaging allows the rapid, noninvasive, and cost-effective assessment of aging and presents a critical advantage in population-scale studies (Li et al., 2022). Recent advances in deep learning, particularly with convolutional neural networks, have revealed the potential to rapidly and accurately predict biological age based on retinal images (Grimbly et al., 2024). Additionally, advances in foundation model technology have further increased the accuracy of retinal age prediction, decreased training data volume, and reduced associated computational expenses (Zhou et al., 2023). The retinal age gap, that is, the difference between predicted retinal age and chronological age, has emerged as a reliable and promising indicator for quantifying aging acceleration (Grimbly et al., 2024). A positive retinal age gap indicates accelerated retinal aging (exceeding chronological age), whereas a negative retinal age gap signifies slower retinal aging, and the retinal age gap is correlated with mortality risk and diseases (e.g., Parkinson’s disease, cardiovascular disease, kidney failure, and diabetic retinopathy) (Zhu et al., 2023; Hu et al., 2022; Zhu et al., 2022; Zhang et al., 2023; Chen et al., 2023a). Thus, retinal age may provide new insight into GGT-related effects on aging. Although previous studies reported established roles of GGT in systemic aging and validated the retinal age gap as an available indicator of aging, the relationship between GGT levels and the retinal age gap remains unexplored. Therefore, this community-based study aimed to investigate the association between GGT levels and the retinal age gap using the foundation model of color fundus photography.

## Materials and methods

### Study design and population

This study was a part of the Jidong Eye Cohort Study (JECS). All the data that were analyzed in this study were collected from participants who were enrolled in the JECS. The detailed design and methodology of the JECS have been previously published (Yang et al., 2020). From August 2019 to October 2023, approximately 10,000 participants were recruited from the Jidong communities (Tangshan, Hebei, China). All the participants were subjected to comprehensive ophthalmic examinations, physical measurements, and biological sample collection, and all the participants completed detailed healthcare questionnaires. This study was approved by the Ethics Committee of the Staff Hospital of Jidong Oil-field of Chinese National Petroleum (approval number: 2018 YILUNZI 1) and the Ethics Committee of the Eye Hospital of Wenzhou Medical University (approval number: 2021-074-K-63-01). The study followed the guidelines of the Declaration of Helsinki, and all the participants provided written informed consent.

### Assessment of color fundus photography

In this study, digital fundus images were captured using a 45° nonmydriatic fundus camera (CR2AF; Canon; Tokyo, Japan) without pupil dilation. A total of 43,558 images from 9,752 participants were collected from the JECS. After quality control, 36,044 images from 9,285 participants met the required standards. The quality control process, described in detail in previous publications (Wang J. et al., 2024; Zhou et al., 2022; 2023), utilized a collaboration between ophthalmologists and an automated retinal image analysis tool that included image quality grading. Color fundus photography with retinal disease (i.e., nerve fiber layer defects, abnormal cup-to-disco ratio, macular degeneration, retinal vein occlusion, diabetic retinopathy, and severe opacification of the refractive media) were ruled out, and only images that were classified as good or usable were considered acceptable for this study.

### Fine-tuning the foundation model for age prediction

Following previous studies (Zhu et al., 2023; Grimbly et al., 2024; Wang J. et al., 2024), chronological age was assumed to match biological age in normally aging individuals. To establish a reliable reference for biological age prediction, the healthy dataset for model training and validation included JECS participants without clinical diagnoses of hypertension, diabetes, chronic kidney disease, cardiovascular disease, or stroke. Subclinical cases were excluded via imaging examinations, physical examinations, and laboratory tests (e.g., fasting blood glucose), ensuring only individuals with normal results were included.

**FIGURE 1:**
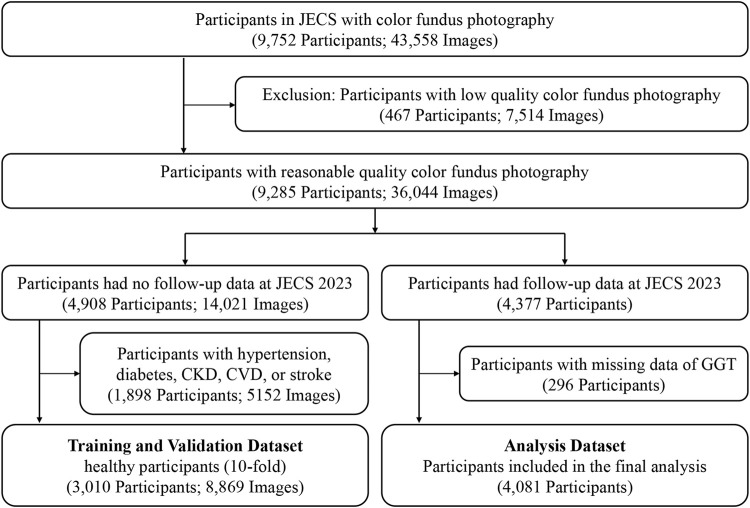
Flow Chart of the Study Participants and Images. JECS, Jidong Eye Cohort Study; CKD, chronic kidney disease; CVD, cardiovascular disease; GGT, gamma-glutamyl transferase.

In this study, all the data were divided into two parts: the data from JECS 2023 were utilized as the analysis dataset only, and the data from JECS 2019 to JECS 2021 were used as the training and validation dataset. To prevent leakage in the analysis set, all the participants who had follow-up data in 2023 were removed from the training and validation datasets. In detail, among the 9,285 participants with fundus images of acceptable quality, we first identified 4,377 individuals who had follow-up data from 2023 (May to October 2023) in the JECS, and these data were included in the analysis dataset. Fundus images from right eyes were primarily used for retinal age calculation, and left-eye images were substituted when right-eye images were unavailable. A total of 4,081 fundus images of the 4,081 participants had available GGT data, and these images were used to investigate the association between GGT levels and the retinal age gap (images and other data were all acquired from May to October 2023). For the remaining 4,908 participants, a total of 8,869 fundus images from 3,010 healthy individuals were selected to form the healthy dataset for model training and validation. This dataset was further divided using 10-fold cross-validation to ensure robust model development (Figure 1).

**FIGURE 2:**
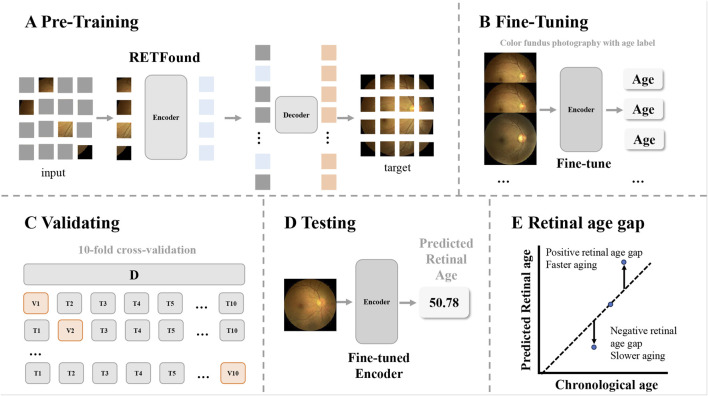
Overview of the study workflow. The workflow illustrates how retinal age gaps were calculated from the color fundus images. **(A)** The pretrained foundation model of color fundus photography used in this study was a Masked Autoencoders architecture. **(B)** The encoder from RETFound was fine-tuned using fundus images of healthy participants, with chronological age as the target (using a linear head). **(C)** Model validation was performed using 10-fold cross-validation. **(D)** The fine-tuned model predicted retinal ages for participants in the analysis dataset. **(E)** The retinal age gap was defined as the difference between predicted retinal age and chronological age, with positive values indicating faster aging and negative values indicating slower aging.

We fine-tuned the foundation model of color fundus photography RETFound (Zhou et al., 2023), which is a state-of-the-art architecture that was pretrained on large-scale retinal image datasets (904,170 unlabeled retinal fundus images), to develop and validate the model for age prediction (Figure 2). Briefly, all the fundus images were preprocessed by resizing to a resolution of 224 × 224 pixels and normalized using the mean and standard deviation of the retinal image dataset. Data augmentation, including random erasing and DeiT-style random augmentation, was applied during training (Zhou et al., 2023). The model was optimized using the L2 loss between the predicted and chronological ages. Training utilized the AdamW optimizer with a 10-epoch warm-up and a cosine learning rate decay policy with an initial learning rate of 0.001. When implemented on an NVIDIA 4090 GPU with a batch size of 64, the model was trained for 100 epochs using PyTorch. Performance was evaluated using the mean absolute error (MAE), mean absolute percentage error (MAPE), root mean squared error (RMSE), and R^2^ between the predicted retinal age and chronological age.

### Definition of the retinal age gap

The retinal age gap was calculated as the difference between the predicted retinal age according to the fundus images and the chronological age. A positive retinal age gap indicated faster retinal aging than chronological age, while a negative retinal age gap suggested slower retinal aging.

### Assessment of general variables

In this study, we collected the participants’ basic information through clinical examinations, laboratory tests, and standardized questionnaires about their demographic features, current smoking and alcohol consumption statuses, and medical history (Su et al., 2022). The average monthly income was classified into two levels: “< ¥3000” and “≥ ¥3000”. Education level was defined as “illiterate, primary or middle school” and “college graduate and above”. In this study, diabetes was defined as a fasting blood glucose (FBG) level ≥7.0 mmol/L, self-reported history of diabetes, or current use of antidiabetic drugs. Hypertension was defined as a systolic blood pressure ≥140 mmHg, diastolic blood pressure ≥90 mmHg, self-reported history of hypertension, or current use of antihypertensive medications. Dyslipidemia was defined as low-density lipoprotein (LDL-C) ≥ 3.3 mmol/L, high-density lipoprotein (HDL-C) < 1.04 mmol/L, total cholesterol (TC) ≥ 5.18 mmol/L, triglyceride (TG) ≥ 1.7 mmol/L, the use of lipid-lowering medications, or a self-reported history of dyslipidemia.

### Assessment of gamma-glutamyl transferase

Fasting venous blood samples were obtained from the elbow in the morning after the participants had fasted from food and drink for at least 8 h, and the samples were stored in vacuum tubes containing ethylenediaminetetraacetic acid. The levels of GGT and serum uric acid (SUA) were measured by an autoanalyzer (Hitachi, Tokyo, Japan) via the uricase‒peroxidase method at the Central Laboratory of Jidong Oil-Field Hospital (Tang et al., 2021). The participants in this study were stratified into quartiles based on the GGT levels (Q1: ≤16 U/L; Q2: 17–23 U/L; Q3: 24–37 U/L; and Q4: ≥38 U/L).

### Statistical analysis

Continuous variables are presented as means and standard deviations (SD), whereas categorical variables are presented as frequencies and percentages. To analyze the differences among different GGT quartile groups, we applied a one-way ANCOVA test for normally distributed continuous variables. For categorical variables, we used chi-square tests or Fisher’s exact tests.

Multivariable generalized linear models were used to assess the relationship between GGT quartiles and the retinal age gap. We treated the GGT quartiles as a continuous ordinal variable to test for trends. The multivariable generalized linear models were adjusted for different sets of covariates: age and sex (Model 1); age, sex, current smoking status, current drinking status, body mass index (BMI), hypertension, diabetes, dyslipidemia, and SUA (Model 2). Furthermore, we examined the associations of a 1 standard deviation change in GGT level with the retinal age gap. Additionally, by adding interaction terms in the adjusted models, we investigated whether the associations between GGT and the retinal age gap varied according to sex, hypertension, diabetes, dyslipidemia, and current drinking status.

The associations are expressed as βs and 95% confidence intervals (CIs). In generalized linear models, βs estimate the absolute change in retinal age gap: for GGT quartiles, they represent the difference relative to the lowest quartile, while for continuous GGT, they reflect the change per 1 SD increase. A positive β indicates an increase in the retinal age gap, and a negative β indicates a decrease. In all the analyses, statistical significance was set to a 2-tailed P value <0.05. All the statistical analyses were carried out using SAS software (version 9.4; SAS Institute Inc., Cary, NC, United States).

## Results

### Performance of the trained model for predicting age

The model was trained and validated on data from 3,010 healthy participants; these participants had a mean age of 40.6 ± 10.9 years at baseline, and 1773 (58.9%) were female. As shown in Table 1, the trained model in this study achieved strong performance on the healthy dataset, with an MAE of 2.42 ± 0.08 years, an RMSE of 3.17 ± 0.10, and an R^2^ of 0.89 ± 0.01; thus, this model is better than those described in previous studies (Ahadi et al., 2023; Zhu et al., 2023; Grimbly et al., 2024; Wang J. et al., 2024).

**TABLE 1:** Performance of the trained model in this study.

10-Fold	MAPE	MAE	RMSE	MSE	R^2^
Fold 1	0.062	2.33	3.10	8.67	0.90
Fold 2	0.065	2.54	3.35	10.10	0.87
Fold 3	0.063	2.36	3.33	9.75	0.87
Fold 4	0.061	2.39	3.05	9.43	0.90
Fold 5	0.064	2.41	3.22	9.55	0.90
Fold 6	0.061	2.37	3.09	8.47	0.89
Fold 7	0.061	2.37	3.10	9.75	0.90
Fold 8	0.063	2.57	3.17	10.31	0.89
Fold 9	0.062	2.41	3.14	9.38	0.89
Fold 10	0.060	2.44	3.13	9.43	0.89
Mean	0.062	2.42	3.17	9.48	0.89
SD	0.002	0.08	0.10	0.57	0.01

MAPE, mean absolute percentage error; MAE, mean absolute error; RMSE, root mean squared error; MSE, mean square error; SD, standard deviations.

### Baseline characteristics of the participants included in the analysis dataset

A total of 4,081 participants from the Jidong communities, who were recruited between May and October 2023, were ultimately included in the analysis. The mean age of the included participants was 43.7 ± 10.4 years, 1987 (48.7%) were female, and the mean retinal age gap was −0.5 ± 4.18 years. Table 2 summarizes the baseline characteristics of the participants in different quartiles of GGT levels. Participants with higher GGT levels were more likely to be male (P < 0.001), current smokers (P < 0.001), and current drinkers (P < 0.001). They were also more likely to have a higher BMI (P < 0.001), higher SUA (P < 0.001), and a higher prevalence of hypertension (P < 0.001), diabetes (P < 0.001), and dyslipidemia (P < 0.001).

**TABLE 2:** Baseline characteristics of participants grouped according to gamma-glutamyl transferase quartiles.

Characteristics	Total (n = 4081)	GGT				P	P For trend
		Q1 (n = 1177)	Q2 (n = 948)	Q3 (n = 976)	Q4 (n = 980)		
Age, y, mean (SD)	43.7 (10.4)	42.5 (10.8)	44.6 (11.0)	44.9 (10.3)	43.0 (9.2)	<0.001	0.08
Retinal age gap, y, mean (SD)	−0.50 (4.18)	−0.76 (4.08)	−0.67 (4.13)	−0.60 (4.4)	0.06 (4.0)	<0.001	<0.001
Sex, n (%)						<0.001	<0.001
Male	2094 (51.3)	176 (15.0)	425 (44.8)	685 (70.2)	808 (82.5)		
Female	1987 (48.7)	1,001 (85.1)	523 (55.2)	291 (59.8)	172 (17.6)		
Educational level, n (%)						0.09	0.3
literacy/Primary Middle School	1,125 (27.6)	300 (25.5)	270 (28.5)	294 (30.1)	261 (26.6)		
College/University	2,956 (72.4)	877 (74.5)	678 (71.5)	682 (69.9)	719 (73.4)		
Income, n (%)						0.2	0.4
<¥5,000	3093 (75.8)	893 (75.9)	723 (76.3)	756 (77.5)	721 (73.6)		
≥¥5,000	988 (24.2)	284 (24.1)	225 (23.7)	220 (22.5)	259 (26.4)		
Current Smoking, n (%)	891 (23.1)	52 (4.7)	148 (16.5)	273 (29.8)	418 (44.7)	<0.001	<0.001
Current Drinking, n (%)	996 (25.8)	61 (5.5)	160 (17.9)	304 (33.2)	471 (50.3)	<0.001	<0.001
Hypertension, n (%)	948 (23.2)	142 (12.1)	190 (20.0)	294 (30.1)	322 (32.9)	<0.001	<0.001
Diabetes, n (%)	359 (8.8)	23 (2.0)	64 (6.7)	105 (10.8)	167 (17.0)	<0.001	<0.001
Dyslipidemia, n (%)	2,562 (62.8)	451 (38.3)	551 (58.1)	713 (73.1)	847 (86.4)	<0.001	<0.001
SBP, mmHg, mean (SD)	123.4 (18.0)	116.4 (17.0)	122.3 (16.9)	126.5 (17.8)	129.5 (17.3)	<0.001	<0.001
DBP, mmHg, mean (SD)	75.5 (12.1)	70.3 (10.6)	74.4 (10.9)	77.5 (11.9)	80.7 (12.2)	<0.001	<0.001
FBG, mmol/L, mean (SD)	5.7 (1.3)	5.2 (0.6)	5.5 (1.1)	5.8 (1.5)	6.1 (1.7)	<0.001	<0.001
BMI, kg/m^2^, mean (SD)	24.9 (3.9)	22.5 (2.8)	24.4 (3.5)	25.8 (3.6)	27.2 (3.9)	<0.001	<0.001
TC, mmol/L, mean (SD)	5.0 (0.9)	4.8 (0.8)	4.9 (0.9)	5.0 (0.9)	5.3 (1.0)	<0.001	<0.001
Triglycerides, mmol/L, mean (SD)	1.9 (1.7)	1.1 (0.7)	1.5 (1.0)	2.1 (1.7)	3.0 (2.4)	<0.001	<0.001
HDL-C, mmol/L, mean (SD)	1.4 (0.3)	1.5 (0.3)	1.3 (0.3)	1.3 (0.3)	1.3 (0.3)	<0.001	<0.001
LDL-C, mmol/L, mean (SD)	2.6 (0.7)	2.4 (0.6)	2.6 (0.7)	2.7 (0.7)	2.9 (0.8)	<0.001	<0.001
SUA, umol/L, mean (SD)	362.3 (94.5)	297.8 (66.0)	347.5 (76.6)	393.0 (86.1)	423.4 (95.0)	<0.001	<0.001
GGT, U/L, mean (SD)	32.6 (32.1)	13.1 (2.1)	19.8 (2.0)	29.6 (4.0)	71.2 (46.4)	<0.001	<0.001
GGT range, U/L	6–422	6–16	17–23	24–37	38–422		

Data were presented as number (percentage) for category variables and mean (SD) for continuous variables.

P for trend tested by considering the GGT, quartile as continuous ordinal variables.

Q1, quartile 1 (n = 1,177): ≤16 U/L; Q2, quartile 2 (n = 948): 17–23 U/L; Q3, quartile 3 (n = 976): 24–37 U/L; Q4, quartile 4 (n = 980): >37 U/L.

GGT, gamma-glutamyl transferase; SBP, systolic blood pressure; DBP, diastolic blood pressure; FBG, fasting blood glucose; BMI, body mass index; TC, total cholesterol; HDL-C, high-density lipoprotein cholesterol; LDL-C, low-density lipoprotein cholesterol; SD, standard deviation; SUA, serum uric acid.

Table 2 also presents the retinal age gap in different GGT quartile groups for the baseline characteristics. The retinal age gap increased from −0.76 years in Q1 to 0.06 years in Q4 (P for trend <0.001).

## Associations between GGT levels and the retinal age gap

The associations between GGT quartiles and the retinal age gap are shown in Table 3. The participants in the highest GGT quartile showed a significant increase in the retinal age gap (β = 1.00, 95% CI = 0.63–1.36, P < 0.001), even after fully adjusting for potential confounding factors. Moreover, compared with those for the participants in the lowest quartile, the fully adjusted βs and 95% CIs for the participants in the second, third, and fourth GGT quartiles were 0.42 (0.08–0.77), 0.54 (0.15–0.92), and 0.72 (0.29–1.14), respectively. Additionally, a significant trend (P for trend ≤0.001) was observed across all quartiles in both models.

**TABLE 3:** Association between gamma-glutamyl transferase levels and the retinal age gap.

GGT (U/L)	β 95%CI	P	P For trend
Model 1			<0.001
Q1	Reference		
Q2	0.51 (0.18–0.84)	0.002	
Q3	0.66 (0.31–1.01)	<0.001	
Q4	1.00 (0.63–1.36)	<0.001	
Model 2			0.001
Q1	Reference		
Q2	0.42 (0.08–0.77)	0.02	
Q3	0.54 (0.15–0.92)	0.007	
Q4	0.72 (0.29–1.14)	0.001	

Model 1: adjusted for age and sex; Model 2: adjusted for age, sex, current smoking status, current drinking status, BMI, hypertension, diabetes, dyslipidemia, and SUA.

βs for quartiles: absolute change vs. Q1.

P for trend tested with generalized linear models by considering the GGT, quartiles as continuous ordinal variables.

Q1, quartile 1 (n = 1,177): ≤16 U/L; Q2, quartile 2 (n = 948): 17–23 U/L; Q3, quartile 3 (n = 976): 24–37 U/L; Q4, quartile 4 (n = 980): >37 U/L.

GGT, gamma-glutamyl transferase; BMI, body mass index; SUA, serum uric acid.

Furthermore, to verify the reliability of our results, we also examined the relationships between a 1 SD change in GGT and the retinal age gap. We found similar relationships. In two models (Model 1: adjusted for age and sex; Model 2: adjusted for age, sex, current smoking status, current drinking status, BMI, hypertension, diabetes, dyslipidemia, and SUA.), the βs values and 95% CIs were 0.31 (0.19–0.43) and 0.19 (0.07–0.33), respectively.

### Subgroup analysis

Subgroup analyses by sex, hypertension, diabetes, dyslipidemia, and current drinking status are presented in Table 4. A significant interaction was found between non-hypertension and continuous GGT quartiles for the retinal age gap. The βs of the continuous GGT quartile items in participants with or without hypertension were 0.18 and 0.22, respectively (P for interaction = 0.04). However, no significant interactions were observed for sex, diabetes, dyslipidemia, or current drinking status with the continuous GGT quartiles for the retinal age gap.

**TABLE 4:** Subgroup analyses of the associations between gamma-glutamyl transferase levels and the retinal age gap were performed on the basis of sex, hypertension, diabetes, dyslipidemia, and current drinking status.

Subgroup	n	β 95%CI	P For interaction
Sex			0.14
Male	2094	0.15 (−0.03–0.34)	
Female	1987	0.36 (0.16–0.56)	
Hypertension			0.04
Hypertension	948	0.18 (−0.18–0.53)	
Non-Hypertension	3133	0.22 (0.08–0.37)	
Diabetes			0.08
Diabetes	359	0.23 (−0.50–0.96)	
Non-Diabetes	3722	0.09 (0.06–0.33)	
Dyslipidemia			0.16
Dyslipidemia	2,562	0.23 (0.05–0.40)	
Non-Dyslipidemia	1,519	0.24 (0.01–0.47)	
Current drinking status			0.93
Current drinkers	996	0.17 (−0.10–0.44)	
Non-current drinkers	2,867	0.27 (0.11–0.43)	

Interaction effect was calculated from models that included interaction terms of factor × continuous GGT, quartiles, and were adjusted for age, sex, current smoking status, current drinking status, BMI, hypertension, diabetes, dyslipidemia, and SUA.

βs for continuous GGT, quartiles: change per 1 quartile.

GGT, gamma-glutamyl transferase; BMI, body mass index; SUA, serum uric acid.

## Discussion

This study provides compelling evidence linking elevated GGT levels with accelerated retinal aging, as quantified by the retinal age gap, in a relatively large community-based population. The multivariable generalized linear model analysis revealed that the participants with GGT levels in the highest quartile had a significantly greater retinal age gap independent of potential confounders, and a significant trend was observed across all quartiles.

In this study, we developed and validated a deep learning model for predicting retinal age using fundus images, and the model achieved strong performance with an MAE of 2.42 years, outperforming most of the previous models in predicting age using fewer training samples. Previous studies have shown MAEs of 2.8–3.5 years for retinal age prediction (Zhu et al., 2023; Ahadi et al., 2023; Chen et al., 2023b; Abreu-Gonzalez et al., 2023), and MAEs of 3.26–3.65 years for multimodal biological age estimation including fundus images (Wang J. et al., 2024). The improvement in the performance and training efficiency of the retinal age prediction model occurred due to the use of the foundation model that was pretrained on 904,170 unlabeled retinal fundus images (Zhou et al., 2023). After being pretrained on a large-scale retinal image dataset, the foundation model learned how to detect and analyze the characteristics of retinal images. Thus, we only needed to fine-tune the model on a relatively small dataset to achieve even better performance (Sevgi et al., 2025).

To the best of our knowledge, this study represents one of the first attempts to demonstrate the concept of an association between GGT levels and the retinal age gap. In our study, we found that higher GGT levels are associated with accelerated retinal aging, as quantified by the retinal age gap. Several previous studies have demonstrated that elevated GGT levels are associated with several age-related diseases, which support our findings. For example, Cho et al. (Cho et al., 2014, pp. 2010–2011; Lei et al., 2020; Kim et al., 2022) previously reported an association between elevated GGT levels and the risk of age-related ocular diseases, including age-related macular degeneration (AMD), primary glaucoma, and ocular motor cranial nerve palsy. Moreover, GGT was proven to be associated with the risk of cardiovascular disease (Kunutsor et al., 2015), Alzheimer’s disease (Kunutsor and Laukkanen, 2016; Kunutsor et al., 2018), Parkinson’s disease (Yoo et al., 2020), and metabolic abnormalities (Ya et al., 2017). Our study results may help explain why people with higher GGT levels are more likely to have age-related diseases. The accelerated aging of participants with higher GGT levels may be explained by the potential proinflammatory and pro-oxidative effects of GGT (Lee and Jacobs, 2005; Turgut et al., 2006; Turgut and Tandogan, 2011; Sarli et al., 2013). In addition, GGT levels are directly involved in the formation of atheromatous plaques, which have been implicated in the mechanism underlying the pathogenesis of vascular aging (Franzini et al., 2009).

In addition, some prior studies have explored the mediating effect of GGT in the aging process, which is supportive of our findings. For example, Liu et al. (Liu et al., 2024, pp. 2005–2018) showed that GGT, along with bilirubin and uric acid, collectively mediated the relationship between Life’s Essential 8 scores and PhenoAgeAccel, which was measured using clinical laboratory blood chemistries. Moreover, two studies reported that GGT plays an important role in the association of sleep and blood benzene with accelerated aging (Wang X. et al., 2024; Yang et al., 2024). Another study using data from the United Kingdom Biobank revealed that the relationships between plant protein and four biological aging measures were mediated by GGT (Xu et al., 2024). The results of these previous studies suggest that GGT has a crucial effect on the aging process, and our findings directly implicate GGT as an independent driver of biological aging.

Our study revealed a stronger association between GGT levels and the retinal age gap in non-hypertensive participants. The associations of GGT levels with the retinal age gap remained consistent across sex, diabetes, dyslipidemia, and current drinking status subgroups. These findings suggest that the absence of hypertension may enhance the association of GGT levels with the retinal age gap. However, further studies are needed to confirm this interplay.

Our findings have several significant implications for public health and clinical practice. First, we investigated the relationship between high GGT levels and accelerated retinal aging, as qualified by the retinal age gap. Our findings extend the utility of GGT beyond its traditional hepatobiliary applications. Second, the retinal age gap, as quantified through advanced imaging analytics, provides a novel framework for assessing aging processes that is low-cost, widely available, noninvasive, and time efficient. Third, our study further shows the advantages of fine-tuning based on the foundation model. This approach not only improves performance but also further reduces training costs.

The main strengths of this study were the process of capturing high-quality fundus images and performing detailed blood biochemical examinations in a relatively large community-based study, adjustments that were made for several potential confounding factors, including lifestyle and metabolic confounders, and the use of sensitivity analyses and subgroup analyses to ensure the robustness of the results. However, several limitations in our study should also be acknowledged. First, the cross-sectional design precludes causal inference. Although we propose that elevated GGT levels drive retinal aging through oxidative pathways, reverse causation remains theoretically possible. Longitudinal studies tracking GGT trajectories and retinal aging progression are needed to establish temporal relationships, and a future study objective may include further follow-up to the JECS. Second, the study participants were all from the Jidong community, and validation in diverse populations is needed to confirm the universal applicability of these findings. Finally, some potential confounders, such as hormone levels and cell factors that may affect GGT levels, the retinal age gap, and residual confounders, were not included in the analysis.

## Conclusion

In conclusion, this study revealed that higher GGT levels were associated with accelerated retinal aging, as quantified by the retinal age gap. Given the accelerating aging of the global population, our findings emphasize that GGT, which serves as a potential systemic biomarker for oxidative stress, may exert a detrimental effect on the aging process, thereby highlighting the importance of integrating GGT assessment into evaluations related to aging.

## Data Availability

The raw data supporting the conclusions of this article will be made available by the authors, without undue reservation.

## Abbreviations

GGT, gamma-glutamyl transferase; JECS, Jidong Eye Cohort Study; MAE, mean absolute error; MAPE, mean absolute percentage error; RMSE, root mean squared error; FBG, fasting blood glucose; LDL-C, low-density lipoprotein; HDL-C, high-density lipoprotein; TC, total cholesterol; TG, triglyceride; SUA, serum uric acid; SD, standard deviation; BMI, body mass index; CIs, confidence intervals; AMD, age-related macular degeneration.
